# A Novel View on the Role of Intracellular Tails in Surface Delivery of the Potassium-Chloride Cotransporter KCC2

**DOI:** 10.1523/ENEURO.0055-17.2017

**Published:** 2017-07-21

**Authors:** Perrine Friedel, Anastasia Ludwig, Christophe Pellegrino, Morgane Agez, Anass Jawhari, Claudio Rivera, Igor Medina

**Affiliations:** 1 INMED, Aix-Marseille University, INSERM, Marseille 13273, France; 2 Neuroscience Center, University of Helsinki, Helsinki 00100, Finland; 3 CALIXAR, Lyon 69008, France

**Keywords:** Chloride, GABA, KCC2

## Abstract

A plethora of neurological disorders are associated with alterations in the expression and localization of potassium-chloride cotransporter type 2 (KCC2), making KCC2 a critical player in neuronal function and an attractive target for therapeutic treatment. The activity of KCC2 is determined primarily by the rates of its surface insertion and internalization. Currently the domains of KCC2 dictating its trafficking and endocytosis are unknown. Here, using live-cell immunolabeling and biotinylation of KCC2 proteins expressed in murine neuroblastoma N2a cells, human embryonic kidney 293 cells, or primary cultures of rat hippocampal neurons, we identified a novel role for the intracellular N and C termini in differentially regulating KCC2 surface expression. We report that the N terminus is required for KCC2 insertion into the plasma membrane, whereas the C terminus is critical for the membrane stability of KCC2. Our results provide novel insights into the structure–function role of specific KCC2 domains and open perspectives in exploring structural organization of this protein.

## Significance Statement

The neuronal potassium-chloride cotransporter KCC2 is critically involved in numerous neurologic disorders. However, the structural components that regulate KCC2 activity remain to be elucidated. Here, we describe novel and differential roles for the intracellular N and C terminal domains of KCC2 that dictate its plasmalemmal insertion and surface stabilization. Our findings challenge the current view on the structure–function role of the cytoplasmic regions of KCC2 and propose new targets in the search for therapeutic treatments.

## Introduction

A low intracellular chloride concentration ([Cl^–^]_i_) is required for inhibitory synaptic transmission in mature neurons and is maintained by neuron-specific potassium-chloride cotransporter type 2 (KCC2) which facilitates the export of chloride ions (Rivera et al., 1999). Decreased KCC2 activity is linked to the etiology of several neurologic disorders including epilepsy, acute trauma, ischemia, postsurgery complications, and neuropathic pain (Blaesse et al., 2009; Kahle et al., 2013; Medina et al., 2014). In both physiologic and pathologic conditions, the major mechanism controlling KCC2 function relies on the control of its surface expression (Chamma et al., 2012; Kahle et al., 2013; Medina et al., 2014). Although recent studies have identified a number of KCC2 amino acid residues involved in its internalization, the domains of KCC2 that regulate its insertion into the plasma membrane remain unknown (Lee et al., 2007, 2010; Kahle et al., 2014; Puskarjov et al., 2014; Weber et al., 2014; Friedel et al., 2015; Stödberg et al., 2015).

The mammalian *KCC2* gene (aka *Slc12a5*) generates two major isoforms, KCC2a and KCC2b, that differ in their N termini (Uvarov et al., 2007) and their developmental and spatial profile of expression (Markkanen et al., 2014). Historically, the first KCC2 molecule, discovered by Payne et al. in 1996, was actually the isoform KCC2b; these days, it is considered the major and most important isoform in the adult brain and spinal cord (Payne et al., 1996; Rivera et al., 1999; Stein et al., 2004; Markkanen et al., 2014). Mice with knocked out KCC2b are characterized by increased epileptiform-like neuronal network activity and die at juvenile age of 2–3 postnatal weeks (Woo et al., 2002). The KCC2a isoform is expressed in immature neurons of all brain regions but is absent or expressed at a low level in mature cortex, hippocampus, thalamus, and cerebellar cortex (Uvarov et al., 2007; Markkanen et al., 2014). Mice with knocked out KCC2a appear grossly normal, although their phenotype has not been characterized in detail (Markkanen et al., 2014). Interestingly, a recent study reported the identification of 11 additional alternative transcripts of KCC2 that are potentially associated with psychiatric disorders in humans; however, their expression profile and functional importance have not been studied yet (Tao et al., 2012). Hydrophobicity analysis of KCC2b amino acid sequence predicted a 2D structure composed of 12 transmembrane domains flanked by intracellular/cytoplasmic N and C termini (Payne et al., 1996). The putative transmembrane region of KCC2 is composed of 532 amino acids, and N and C termini count 102 and 482 amino acids, respectively (Payne et al., 1996).

Here, we identified the region on KCC2b isoform that contributes to the surface insertion of the molecule. Using live-cell immunolabeling, we analyzed the surface expression of KCC2b proteins harboring an extracellular pHluorin tag (KCC2-pH_ext_) and missing either intracellular N or C terminus. We found that the deletion of the N terminal part of KCC2-pH_ext_ (ΔNTD-KCC2-pH_ext_) altered the delivery of the transporter into the plasma membrane of cultured hippocampal neurons, neuron-derived cell line (N2a), and human embryonic kidney 293 (HEK293) cells. Conversely, the construct composed of the N terminus portion and 12 transmembrane domains only—i.e., deletion of the entire intracellular C tail (ΔCTD-KCC2-pH_ext_)—was effectively inserted into the plasma membrane. The compromised surface expression of ΔNTD-KCC2-pH_ext_ was not an artifact of the extracellular tag, as biotinylation of N2a cells overexpressing nontagged constructs confirmed the effective surface labeling of wild-type KCC2 and ΔCTD-KCC2, but not ΔNTD-KCC2 mutant. Our results identify the KCC2 N terminus as a region indispensable for plasma membrane insertion of the transporter and suggest that the large C terminus tail is not critical for this process, but rather plays a role in KCC2 stabilization at the cell surface.

## Materials and Methods

All manipulations with animals were performed in agreement with the guidelines of the Animal Care and Use Committee of INSERM.

### Expression constructs

KCC2-IRES-GFP (Chudotvorova et al., 2005), eGFP-KCC2 (Pellegrino et al., 2011), and KCC2-pH_ext_ (Kahle et al., 2014) were provided by I. Medina. ΔNTD-KCC2 (Li et al., 2007) was obtained from C. Rivera. Nontagged wild-type KCC2 (WT-KCC2) was obtained by removal of the sequence encoding the eGFP tag from eGFP-KCC2. ΔNTD-KCC2-pH_ext_ mutant (deletion of 1–100 amino acids) was created using PCR. ΔCTD-KCC2 mutant (deletion of KCC2’s 654–1114 amino acids) was made by deleting the corresponding piece of KCC2 using BamHI endonuclease. mCherry construct was created by insertion of an ubiquitin promoter instead of CMV promoter in pmCherry vector (Clontech). All constructs were verified by DNA sequencing. More details on vectors and constructs are available on request.

### Primary cultures and transfection of rat hippocampal neurons

Hippocampal neurons from 18-d-old Wistar rat embryos of either sex were dissociated using trypsin and plated onto polyethyleneimine-coated coverslips at a density of 70,000 cells/cm^2^ in minimal essential medium (MEM) supplemented with 10% NU serum (BD Biosciences), 0.45% glucose, 1 mm sodium pyruvate, 2 mm glutamine, and 10 IU/ml penicillin/streptomycin. On day 9 of culture incubation, half of the medium was changed to MEM with 2% B27 supplement (Invitrogen). Transfection of cultured neurons was performed as described by Buerli et al. (2007) and briefly summarized below. 300 µl Opti-MEM medium was mixed with 7 µl Lipofectamine 2000 (Invitrogen), 1 µl Magnetofection CombiMag (OZ Biosciences), and 1.5 µg premixed DNAs encoding constructs of interest. The mixture was incubated for 20 min at room temperature and thereafter distributed dropwise above the neuronal culture. Culture dishes were placed on a magnetic plate (OZ Biosciences) and incubated for 40 min at 37°C, 5% CO_2_. Transfection was terminated by the substitution of 70% of the incubation solution with fresh culture medium. Cells were used in the experiments 3 d after transfection.

### Culture and transfection of N2a and HEK293 cells

Mouse neuroblastoma cells (N2a; ATCC, #CCL-131) and HEK293 cells (ATCC, #CRL-1573) were cultured in Dulbecco’s modified Eagle’s medium supplemented with 10% FBS and 10 IU/ml penicillin/streptomycin. The cells were transfected with appropriate pcDNAs using Lipofectamine 2000 (Invitrogen) according to the manufacturer’s protocol and used 48–72 h after transfection.

### Antibodies for immunocytochemistry

Primary antibodies used for immunocytochemistry were rabbit polyclonal anti-GFP (dilution 1:500; Invitrogen), mouse monoclonal anti-GFP (dilution 1:600; Novus Biologicals), and rabbit polyclonal anti-DsRed (dilution 1:500; Takara Bio). Secondary antibodies were Cy3-conjugated goat anti-rabbit IgG (dilution 1:500; Jackson ImmunoResearch Laboratories), Alexa Fluor 488–conjugated goat anti-mouse IgG (dilution 1:500; FluoProbes), and Alexa Fluor 647–conjugated goat anti-rabbit IgG (dilution 1:500; EMD Millipore). Cell nuclei were revealed using 5-min staining with Hoechst 33258 (1 µg/ml, Sigma-Aldrich).

### Gramicidin perforated patch clamp recording

For estimation of [Cl^-^]_i_ in N2a cells, the cells were transfected with a mixture of two mammalian expression constructs encoding the human α1 subunit of the glycine receptor (GlyR) and KCC2-pH_ext_, eGFP (mock), or KCC2-IRES-GFP (KCC2). For [Cl^-^]_i_ measurements in cultured neurons, 6 d *in vitro* (DIV) neurons were transfected with KCC2-pH_ext_, eGFP (mock), or KCC2-IRES-GFP (KCC2). Measurements were performed 2 or 3 d after transfection (corresponding to 8 or 9 DIV neurons). Coverslips with N2a cells or neurons were placed onto the inverted microscope and perfused with an external solution (in mm): 140 NaCl, 2.5 KCl, 20 Hepes, 20 d-glucose, 2.0 CaCl_2_, 2.0 MgCl_2_, and 0.02 Bumetanide, pH 7.4. For recording from neurons, external solution contained 0.3 µm strychnine and 1 µm tetrodotoxin. The recording micropipettes (5 MΩ) were filled with a solution containing (in mm): 150 KCl, 10 Hepes, and 20 µg/ml gramicidin A, pH 7.2. Glycine (50 μm, for recordings of N2a cells) or isoguvacine (30 µm, a selective agonist of GABA_A_R for recordings of neurons) was dissolved in external solution and focally applied to recorded cells through a micropipette connected to a Picospritzer (General Valve Corporation, pressure 5 p.s.i.). Recordings were done with an Axopatch-200A amplifier and pCLAMP acquisition software (Molecular Devices) in voltage-clamp mode. Data were low-pass filtered at 2 kHz and acquired at 10 kHz.

### Surface immunolabeling and analysis

Before labeling, half of culture medium was removed from dishes containing cultured neurons or cell lines and transferred to a single centrifuge tube containing polyclonal rabbit anti-GFP antibody. The mixture was centrifuged for 5 min at 8000 rpm, and the supernatant was placed into the cell culture incubator for at least 30 min (to equilibrate with CO_2_ and temperature) and distributed afterward to dishes containing neurons. Neurons exposed to the medium containing primary antibody were kept in the incubator at 37°C for 2 h. The incubation time was determined experimentally to obtain approximately equal amounts of fluorescence intensity emitted by surface located and internalized clusters in neurons expressing WT-KCC2-pH_ext_ and revealed as described below. After labeling, cultures were transferred at room temperature to Hepes-buffered saline and placed for 10 min into the thermo-isolated box at 13°C. The cells were then incubated at 13°C for 20 min with anti-rabbit Cy3-conjugated antibody that revealed the plasma membrane KCC2-pH_ext_ pool (F_m_). The temperature, time, antibody concentration, and secondary antibody [Cy3 AffiniPure Goat Anti-Rabbit IgG (H + L)] were selected to obtain reproducible staining of KCC2-pH_ext_ located on the cell surface (as shown in single plane images of Fig. 3*B*, upper raw). Lowering the temperature to <10°C or increasing the incubation time with Cy3-conjugated antibody to >40 min resulted in damage of the plasma membrane (positive staining of neurons with eGFP-KCC2). Increasing the antibody concentration (dilution 1:200) resulted in an increase of the background fluorescence on nontransfected neurons without improving the brightness and number of clusters on the transfected ones. The staining with other secondary antibodies conjugated with Cy5, Alexa Fluor 546, 633, or 647 fluorophores was less effective. After rinsing in a Hepes-buffered saline solution for 10 min at 13°C, the cells were fixed in Antigenfix (Diapath) for 20 min (room temperature), permeabilized (0.3% Triton X-100), blocked (5% goat serum) for 30 min, and labeled with a second secondary antibody (anti-rabbit Alexa Fluor 647) for 1 h. This staining allowed visualization of labeled internalized proteins (fluorescence signal located inside of the neuron at individual confocal *z*-scans, Fig. 3*B*). The staining with Alexa Fluor 647 antibody also revealed labeled WT-KCC2-pH_ext_ clusters located on the cell surface (i.e., Cy3-positive). This indicated that the epitopes on primary antibodies were not saturated during live-cell staining with Cy3-conjugated antibody or were unmasked during the fixation/permeabilization procedure. Therefore, to dissect the “pure” internalized pool of labeled molecules, the single-plane images of Alexa Fluor 647 and Cy3 emitted fluorescence were treated arithmetically afterward as described below. To detect the total level of ectopic KCC2-related proteins expressed, the cells were finally labeled with mouse anti-GFP antibody (1 h, room temperature) and revealed with anti-mouse Alexa 488 antibody (1 h, room temperature).

To reveal all the clusters labeled on the cell surface (F_all_), we omitted the live-cell staining step with the anti-rabbit Cy3-conjugated antibody and revealed labeled proteins by applying a single secondary anti-rabbit Alexa Fluor 647 antibody to fixed and permeabilized cells.

For quantitative analysis, images of labeled cells were acquired with an Olympus Fluorview-500 confocal microscope using oil-immersion objective 60× (NA 1.4), zoom 3, and 15 pixels/µm image resolution. We randomly selected and focused on a transfected cell by visualizing only Alexa Fluor 488 fluorescence and then acquired *z*-stack images of Alexa Fluor 488, Cy3, and Alexa Fluor 647 fluorochromes emitted fluorescence using, respectively, green (excitation 488 nm, emission 505–525 nm), red (excitation 543 nm, emission 560–600 nm), and infrared (excitation 633, emission >660 nm) channels of the microscope. Each *z*-stack included 10 planes of 1-µm optical thickness and 0.7 µm distance between planes. The cluster properties and fluorescence intensities of each cell were analyzed with MetaMorph software (Molecular Devices Corp.). First, we made subtraction between Alexa Fluor 647 and Cy3 images to isolate in each focal plane the Alexa Fluor 647 signal that was not overlapping with Cy3 fluorescence. This gave rise to additional “internalized pool” images. Second, the arithmetic summation for each *z*-stack and channel was performed to collect the whole fluorescence of the different signals (Alexa Fluor 488; Cy3; internalized pool; Alexa Fluor 647). Third, a binary mask was created for each cell from Alexa Fluor 488 image to isolate the signal coming from the transfected neuron, and the fluorescence parameters (total fluorescence, single cluster fluorescence, and density and brightness of clusters) were analyzed for each channel (Alexa Fluor 488, Cy3, internalized pool, and Alexa 647) in regions overlapping with the binary mask. The analysis parameters were the same for each experiment, and all experiments were done blind.

### Quantification of KCC2 mutant expression in neuronal compartments

13 DIV cultured hippocampal neurons cotransfected with mCherry and KCC2-pH_ext,_ ΔNTD-KCC2-pH_ext_, or ΔCTD-KCC2-pH_ext_ were fixed with Antigenfix 48 h after transfection, then permeabilized and immunolabeled using rabbit anti-DsRed polyclonal antibody and mouse anti-GFP antibody followed by Cy3-conjugated anti-rabbit and Alexa Fluor 488–conjugated anti-mouse. The confocal images of neurons were taken as described in the previous paragraph and analyzed using the following method. For each channel, *z*-stacks of 10 planes were converted into a single image using arithmetic summation with MetaMorph software, and a binary mask was generated based on Cy3-emitted signal. Alexa Fluor 488 fluorescence was measured within the binary mask, normalized to Cy3 intensity, and reported as the total expression of KCC2 protein. Regions of interest (ROIs) were then drawn on the binary mask to identify proximal (40–60 µm from soma) and distal (150–200 µm from soma) dendrites. Alexa Fluor 488 fluorescence was measured in each ROI and normalized to the reference ROI (ref).

### SDS-PAGE and Western blot analysis

N2a cells were plated on 35-mm cell culture dishes 24 h before transfection to obtain 80% confluence at the day of transfection. Transfections with 1.5 µg per dish of DNA encoding KCC2, KCC2-pH_ext_, ΔNTD-KCC2, ΔNTD-KCC2-pH_ext_, ΔCTD-KCC2, ΔCTD-KCC2-pH_ext_, or pcDNA3.1 (mock) were performed using Lipofectamine 2000 according to the manufacturer’s protocol. 36 h after transfection, the cells were gently rinsed with ice-cold PBS complemented with 10 mm iodoacetamide and complete protease inhibitor cocktail (Roche), scraped, and centrifuged for 10 min at 3000 × *g* (4°C). The pellet was dissolved in ice-cold RIPA buffer (150 mm NaCl, 1% Triton X-100, 0.5% deoxycholate, 0.1% SDS, 50 mm Tris-HCl, and 10 mm iodoacetamide, pH 8.0) complemented with complete protease inhibitor cocktail (Roche). The addition of iodoacetamide was critical to reduce the formation of KCC2 containing high-molecular-weight aggregates. Lysates were incubated for 30 min at 4°C with rotation and centrifuged at 1200 × *g* for 5 min to remove debris. Total protein concentrations were determined with the micro BCA protein assay kit (Pierce) using BSA (Sigma-Aldrich) as standard. Same-day lysates were dissolved in Laemmli buffer (2% SDS, 20% glycerol, 5% β-mercaptoethanol, and 62.5 mm Tris HCl,pH 7), preheated to 95˚C, and directly loaded to a SDS-PAGE gel (Bolt 4–12% Bis-Tris Plus precast gels, Thermo Fisher Scientific, 20 μg protein per lane). After transferring the proteins onto a nitrocellulose membrane (Thermo Fisher Scientific), the blots were probed first with chicken anti-KCC2 antibody (KCC2_chk_, dilution 1:4000), recognizing the N terminus of the transporter (Markkanen et al., 2014), and revealed with anti-chicken horseradish peroxidase (HRP)-conjugated antibodies (1:3000, Invitrogen). Thereafter, the secondary antibody was stripped by 3-min incubation at 22°C with Restore PLUS Western Blot Stripping Buffer (Thermo Fisher Scientific), and membranes were probed with a mixture of anti-KCC2 antibody (KCC2_rab_), recognizing the C terminus of the transporter (dilution 1:5000; US Biological, Euromedex) and mouse anti–α-tubulin antibody (α-tub, 1:3000, Thermo Fisher Scientific). The secondary antibodies were anti-rabbit HRP-conjugated (1:3000, Invitrogen) and anti-mouse Cy3-conjugated immunoglobulins (1:2000; Jackson ImmunoResearch Laboratories). The chemiluminescent HRP Substrate (Immobilon, WBKLS0500, EMD Millipore) was used to reveal HRP. All chemiluminescence and fluorescence signals were visualized with G:BOX gel imaging system (Syngene) and Genesys software.

### Membrane biotinylation assay

N2a cells were plated on 60-mm cell culture dishes 24 h before transfection to obtain 80% confluence at the day of transfection. Transfections with 5 µg per dish of DNA encoding KCC2, ΔNTD-KCC2, ΔCTD-KCC2, or pcDNA3.1 (mock) were performed using Lipofectamine 2000 according to the manufacturer’s protocol. 36 h after transfection, the cells were gently rinsed with PBS complemented with 0.1 mm CaCl_2_ and 1 mm MgCl_2_ (PBS/Ca/Mg) and incubated at 20°C with 2 mg/ml EZ-Link Sulfo-NHS-SS-Biotin (21945, Thermo Fisher Scientific) in PBS/Ca/Mg for 30 min. The reaction was quenched using PBS/Ca/Mg plus 100 mm lysine (diluted with water to a final osmolarity of 300 mOsm). The cells were then rinsed three times with PBS/Ca/Mg and lysed in ice-cold RIPA lysis buffer. Lysates were incubated for 30 min at 4°C with rotation and centrifuged at 1200 × *g* for 5 min to remove debris. Total protein concentrations were determined with the micro BCA protein assay kit (Pierce) using BSA (Sigma-Aldrich) as standard. 20 μg of protein were collected and stored overnight at 4°C (to maintain the same temperature regimen as the fraction that was used for biotinylation process). 100 μg protein was incubated with streptavidin-conjugated agarose (20347, Thermo Fisher Scientific) overnight at 4°C with rotation. The next day, the agarose with bound biotinylated membrane proteins was washed three times with ice-cold RIPA buffer and once with ice-cold PBS. Biotin-labeled proteins were eluted in 50 µl Laemmli buffer preheated to 95˚C (2% SDS, 20% glycerol, 5% β-mercaptoethanol, and 62.5 mm Tris HCl, pH 7), and the entire volume was directly loaded to a SDS-PAGE gel. The described protocol includes important details that were experimentally elaborated to decrease the amount of biotin leakage from the intracellular environment. They include gentle rinsing of cells, incubation temperature at 20°C (but not 4°C), and the use of lysine instead of glycine for biotin quenching.

### Analysis of Western blots

In agreement with previous reports (Blaesse et al., 2006; Medina et al., 2014; Friedel et al., 2015), the SDS-PAGE and Western blot analysis of KCC2 and its mutants revealed a multiband pattern of transporter migration. The bottom band reflected migration of the monomeric form of the protein, whereas upper bands were agglomerates presumably containing oligomers and protein complexes formed by interaction with other proteins. The molecular weight of KCC2 and its mutants was estimated based on the migration of the monomeric band, whereas for quantitative analysis of the total protein expression, the intensity of the entire signal (monomer + agglomerates) was taken into account as was previously described (Friedel et al., 2015). As a negative control, we used lysates of cells transfected with pcDNA3.1 (mock). Band intensities were quantified using MetaMorph software.

For the biotinylation experiments, the endogenous transmembrane α-transferrin receptor was used as a loading control for both total cell lysates and biotinylated extracts. The amounts of total and biotinylated loaded fractions were adjusted to obtain approximately similar intensity of WT-KCC2 bands. The intracellular endogenous α-tubulin served as a control of biotin leakiness. The results were quantified as the ratio of biotinylated/total protein for each KCC2-related construct, α-transferrin receptor, and α-tubulin.

### Statistical analysis

Statistical analyses were conducted with OriginPro 9.0.0, which also indicated that assumptions of normality (Shapiro–Wilk test) and equal variance (Brown–Forsythe test) were met. *p* < 0.05 was considered significant for these and all subsequent tests. For data displaying normal distribution, one-way ANOVA and *post hoc* Tukey test were used for multiple comparisons between groups. For data displaying nonnormal distribution, or experiments with a low number of independent replicates (*n* < 7), Mann–Whitney *U* test was used for comparison between two independent groups and Wilcoxon matched pairs test to compare paired data.

## Results

### Cellular expression of KCC2-pH_ext_–related constructs

**Figure 1.**
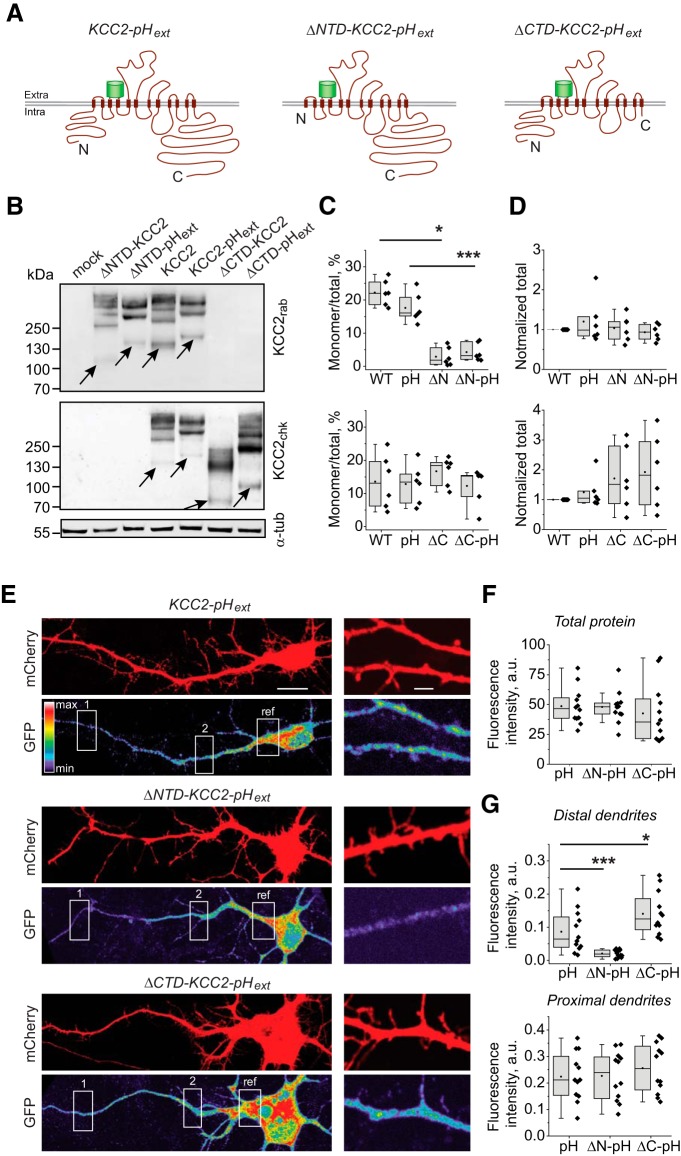
Biochemical properties and expression profile of KCC2-pH_ext_ and its mutants with truncated N and C termini. ***A***, Schematic drawings of KCC2-pH_ext_, ΔNTD-KCC2-pH_ext_, and ΔCTD-KCC2-pH_ext_. The ΔNTD-KCC2-pH_ext_ and ΔCTD-KCC2-pH_ext_ mutants were created by deletion of the first 100 amino acids and the last 470 amino acids from, respectively, the N and C termini of KCC2-pH_ext_. ***B***, Western blot of extracts from N2a cells overexpressing pcDNA3.1 (mock) and indicated KCC2-derived constructs. Arrows indicate the location of the monomer band for each respective construct. The blot was first revealed using anti-KCC2_chk_ antibody recognizing KCC2’s C terminus domain and then using anti-KCC2_rab_ antibody raised against N terminus of KCC2b and anti–α-tubulin antibody. Note the weak intensity of monomer band of ΔNTD-KCC2 and ΔNTD-KCC2-pH_ext_ mutants. ***C***, Tukey boxplots of the calculated monomer/total protein ratio for each construct revealed using anti-KCC2_rab_ (top) and anti-KCC2_chk_ (bottom) antibodies. WT, wild-type KCC2; pH, KCC2-pH_ext_; ΔN, ΔNTD-KCC2; ΔN-pH, ΔNTD-KCC2-pH_ext_; ΔC, ΔCTD-KCC2; ΔC-pH, ΔCTD-KCC2-pH_ext_; six experiments. ***, *p* < 0.001; *, *p* < 0.05; nonparametric Wilcoxon matched pairs test. ***D***, Tukey boxplots of the total expression of KCC2-related constructs normalized to the intensity of endogenous α-tubulin. No statistically significant difference was detected between wild-type KCC2 and each particular mutant. Nonparametric Wilcoxon matched pairs test; six experiments (see Table 1 for the exact *p* values). ***E***, Representative images of neurons transfected with mCherry and mentioned mutants of KCC2-pH_ext_. The mCherry was expressed to visualize the morphology of the neuron and was revealed using rabbit polyclonal anti-DsRed antibody and Cy3-conjugated secondary antibody. The cellular expression of KCC2-pH_ext_ was revealed using mouse anti-GFP antibody and Alexa Fluor 488–conjugated secondary antibody. The fluorescent images of Alexa Fluor 488 were scaled for each neuron to obtain 90% of maximal intensity in the brightest region and were false-colored using rainbow lookup table shown on the left. The white rectangles indicate regions where ROIs were drawn and fluorescence intensities measured for quantification shown in ***G***. The high zoom images were taken at a distance 150–200 µm from soma and illustrate KCC2-pH_ext_ mutants’ expression in secondary dendrites. Scale bars are 20 µm (left) and 1 µm (right). Note that wild-type KCC2-pH_ext_ as well as both mutants were expressed in the soma and dendrites, including the tiny dendrite extremities, but the level of ΔNTD-KCC2-pH_ext_ expression was lower in distal dendrites. ***F***, Quantification of GFP fluorescence intensity (normalized to mCherry) in neurons expressing KCC2-pH_ext_ (pH), ΔNTD-KCC2-pH_ext_ (ΔN-pH), or ΔCTD-KCC2-pH_ext_ (ΔC-pH). Pooled data from three experiments, four neurons per experiment and condition. No statistical differences were observed between conditions, Mann–Whitney *U* test (see Table 1 for details). ***G***, Quantification of GFP fluorescence intensity in distal (top, corresponding to region 1 in ***E***) and proximal (bottom, corresponding to region 2 in ***E***) dendrites of transfected neurons. The data were normalized to the fluorescence of a reference region (ref). Pooled data from three experiments, four neurons per experiment and condition. *, *p* < 0.05; ***, *p* < 0.001, Mann–Whitney *U* test (see Table 1 for details). For the boxplots, the box extends from the first (Q1) to third (Q3) quartiles. The line and solid circle inside the box represent median and mean, respectively. The whiskers define the outermost data point that falls within upper inner and lower inner fence [Q1-1.5(IQR) and Q3-1.5(IQR), respectively]. Black dots show values of individual measurements.

To study the role of KCC2 intracellular domains in cell surface expression of the transporter, we generated KCC2 fusion proteins in which a pHluorin tag was inserted into the second putative extracellular loop of KCC2 protein (KCC2-pH_ext_), and either the N or C terminus of the protein was deleted (ΔNTD-KCC2-pH_ext_ and ΔCTD-KCC2-pH_ext_, respectively; Fig. 1*A*). Tagging a membrane protein is challenging, and problems can occur such as protein instability, misfolding, aberrant posttranslational modifications, and functional changes (Maue, 2007). Thus, we first verified the protein expression level of the KCC2-related constructs. Whole-cell extracts from N2a cells, transiently transfected with KCC2-pH_ext_, ΔNTD-KCC2-pH_ext_, or ΔCTD-KCC2-pH_ext_, were analyzed by Western blotting using denaturing conditions (Figs. 1*B*–*D*). For comparison, we used a nontagged wild-type KCC2 and the respective deleted mutants, ΔNTD-KCC2 and ΔCTD-KCC2.

The upper panel in Fig. 1*B* shows a representative Western blot of ΔNTD-KCC2, ΔNTD-KCC2-pH_ext_, KCC2, and KCC2-pH_ext_, visualized using a polyclonal antibody that recognizes an epitope located in the C terminus region of the transporter (anti-KCC2_rab_). A multiple band migration pattern was detected, in which the bottom band corresponds to the monomeric state of the transporter (Fig. 1*B*, ∼140 kDa, indicated with an arrow), whereas the upper bands account for oligomers or high-molecular-weight protein complexes that were resistant to denaturing conditions (>280 kDa). This migration pattern was described previously and is typical for the KCC2 transporter (Blaesse et al., 2006; Medina et al., 2014; Friedel et al., 2015). The insertion of the pHluorin tag in KCC2 protein (KCC2-pH_ext_) produced an expected 28 kDa increase in the molecular weight of the monomer band and a similar upward shift of the oligomer/protein complex migration (Fig. 1*B*). The pattern of migration, the ratio of monomer to total protein, and the relative amount of total protein expression were similar for KCC2-pH_ext_ and non-tagged KCC2 (Fig. 1*B–D* and Table 1).

**Table 1.** Statistical differences among the samples illustrated in Figures 1 and 2

Location	Data reference	Data structure	Type of test	Power
a	Fig. 1*C*, upper panel	Nonnormal distribution	Wilcoxon matched pairs test	
	WT vs. pH			*z =* 1.68, *n* = 6; *p =* 0.090
	WT vs. ΔN			*z =* 2.09, *n* = 6; *p =* 0.031
	pH vs. ΔN-pH			*z =* 2.09, *n* = 6*; p =* 0.031
	ΔN vs. ΔN-pH			*z =* –0.84, *n* = 6; *p =* 0.840
b	Fig. 1*C*, lower panel	Nonnormal distribution	Wilcoxon matched pairs test	
	WT vs. pH			*z =* 0, *n* = 6; *p =* 1.000
	WT vs. ΔC			*z =* –1.04, *n* = 6; *p =* 0.312
	pH vs. ΔC-pH			*z =* 0, *n* = 6; *p =* 1.000
	ΔC vs. ΔC-pH			*z =* 1.46, *n* = 6; *p =* 0.160
c	Fig. 1*D*, upper panel	Nonnormal distribution	Wilcoxon matched pairs test	
	WT vs. pH			*z =* –0.21, *n* = 6; *p =* 0.840
	WT vs. ΔN			*z =* 0, *n* = 6; *p =* 1.000
	pH vs. ΔN-pH			*z =* 1.25, *n* = 6; *p =* 0.109
	ΔN vs. ΔN-pH			*z =* 1.35, *n* = 6; *p =* 0.093
d	Fig. 1*D*, lower panel	Nonnormal distribution	Wilcoxon matched pairs test	
	WT vs. pH			*z =* –0.63, *n* = 6; *p =* 0.563
	WT vs. ΔC			*z =* –1.26, *n* = 6; *p =* 0.219
	pH vs. ΔC-pH			*z =* –1.26, *n* = 6; *p =* 0.922
	ΔC vs. ΔC-pH			*z =* –1.05, *n* = 6; *p =* 0.891
e	Fig.1 F	Nonnormal distribution	Mann-Whitney *U*-test	
	WT vs. ΔNTD			*U =* 68, *n* = 12,12; *p =* 0.843
	WT vs. ΔCTD			*U =* 93, *n* = 12,12; *p =* 0.242
f	Fig. 1*G*, upper panel	Nonnormal distribution	Mann–Whitney *U* test	
	WT vs. ΔNTD			*U =* 12, *n* = 12,12; *p =* 5.8 × 10^–4^
	WT vs. ΔCTD			*U =* 36, *n* = 12,12; *p =* 0.040
g	Fig. 1*G*, lower panel	Nonnormal distribution	Mann–Whitney *U* test	
	WT vs. ΔNTD			*U =* 72, *n* = 12,12; *p =* 0.976
	WT vs. ΔCTD			*U =* 62, *n* = 12,12; *p =* 0.562
h	Fig. 2*B*	Normal distribution	One-way ANOVA	*F*(2,28) = 118.15, *p =* 2.24 × 10^–14^
	Mock vs. WT		*Post hoc* Tukey	*p =* 1.70 × 10^–13^
	Mock vs. KCC2-pH_ext_		*Post hoc* Tukey	*p =* 3.75 × 10^–13^
	WT vs. KCC2-pH_ext_		*Post hoc* Tukey	*p =* 0.89
i	Fig. 2*C*	Normal distribution	One-way ANOVA	*F*(2,29) = 12.46, *p =* 1.24 × 10^–4^
	Mock vs. WT		*Post hoc* Tukey	*p =* 2.36 × 10^–4^
	Mock vs. KCC2-pH_ext_		*Post hoc* Tukey	*p =* 8.85 × 10^–4^
	WT vs. KCC2-pH_ext_		*Post hoc* Tukey	*p =* 0.95

The deletion of the N terminus region in nontagged KCC2 protein (ΔNTD-KCC2) resulted in an expected downward shift of both the monomer band (predicted molecular weight, 128 kDa) and the oligomer/protein complexes bands compared with wild-type KCC2 (Fig. 1*B*). The insertion of the pHluorin tag in ΔNTD-KCC2 (ΔNTD-KCC2-pH_ext_) provoked a positive shift in the molecular weight of the monomer band (up to 146 kDa; Fig. 1*B*). Remarkably, we observed that in all experiments, the monomer band of ΔNTD-KCC2 (with or without tag) was barely detectable, whereas the majority of ΔNTD-KCC2 proteins were concentrated in high-molecular-weight complexes. Although the total amount of ΔNTD-KCC2 and ΔNTD-KCC2-pH_ext_ protein expression did not differ from that of wild-type KCC2, we quantified a significant decrease in the ratio of monomer to total compared with wild-type KCC2 (Fig. 1*C*, *D* and Table 1). Therefore, the deletion of the N terminus of KCC2 does not perturb protein translation but significantly enhances the ability of the transporter to form multimolecular complexes resistant to denaturing conditions.

The lower panel of Fig. 1*B* illustrates the migration pattern of KCC2, KCC2-pH_ext_, ΔCTD-KCC2, and ΔCTD-KCC2-pH_ext_ visualized on the same Western blot but using a polyclonal antibody that recognizes an epitope localized on the N terminus domain of the transporter (anti-KCC2_chk_). The monomer band of ΔCTD-KCC2 migrated close to the predicted position of 71 kDa (Fig. 1*B*), and the ratio monomer/total protein did not statistically differ from those of wild-type KCC2 (Fig. 1*B*, *C* and Table 1). Although the high-molecular-weight complexes of ΔCTD-KCC2 exhibited smear-like migration patterns whose aspect varied from one experiment to another (compare Figs. 1*B* and 7*B* ), the total intensity of ΔCTD-KCC2 protein did not differ from that of wild-type KCC2 (Fig. 1*B*, *D* and Table 1). The insertion of a pHluorin tag into ΔCTD-KCC2 protein produced an upward shift of both monomer and oligomer/protein complexes (ΔCTD-KCC2-pH_ext_, Fig. 1*B*) with a similar smear-like pattern for the high-molecular-weight complex. The consistency of molecular weight for ΔCTD-KCC2 monomer bands suggests that the mutant was correctly transcribed and translated. However, the deletion of the C terminus formed a smear-like migration pattern for the high-molecular-weight complexes.

Next, to assess the subcellular distribution of created mutants, we transfected cultured hippocampal neurons with a mixture of plasmids encoding KCC2-pH_ext_–derived constructs and mCherry, to visualize the neuron morphology (Fig. 1*D*). Both ΔNTD-KCC2-pH_ext_ and ΔCTD-KCC2-pH_ext_ mutants were expressed in the form of clusters in the soma, proximal, and distal dendrites. The total intensity of fluorescence emitted by ΔNTD-KCC2-pH_ext_ and ΔCTD-KCC2-pH_ext_ neurons and normalized to the fluorescence of mCherry was not statistically different from that of wild-type KCC2-pH_ext_ (Fig. 1*E*, *F* and Table 1). Similarly, there was no statistically significant difference in the relative level of mutants expression in proximal dendrites (Fig. 1*E*, *G* and Table 1). However, the relative amount of protein expression in distal dendrites was twofold lower for ΔNTD-KCC2-pH_ext_ (*p* = 5.8 × 10^–4^, Mann–Whitney *U* test, Fig. 1*E*, *G* and Table 1) and higher for ΔCTD-KCC2-pH_ext_ (*p* = 0.04, Mann–Whitney *U* test, Fig. 1*E*, *G* and Table 1) compared to KCC2-pH_ext_.

In overall, the insertion of a pHluorin tag in the extracellular loop of KCC2 mutants did not disturb the biochemical properties of the proteins compared to their nontagged homologs. Although the deletion of either N or C terminus of KCC2 did slightly perturb the migration pattern of the proteins as well as their distal dendrite localization, the proteins are effectively transcribed, translated, and expressed in neurons.

### Functionality of KCC2-pH_ext_

**Figure 2.**
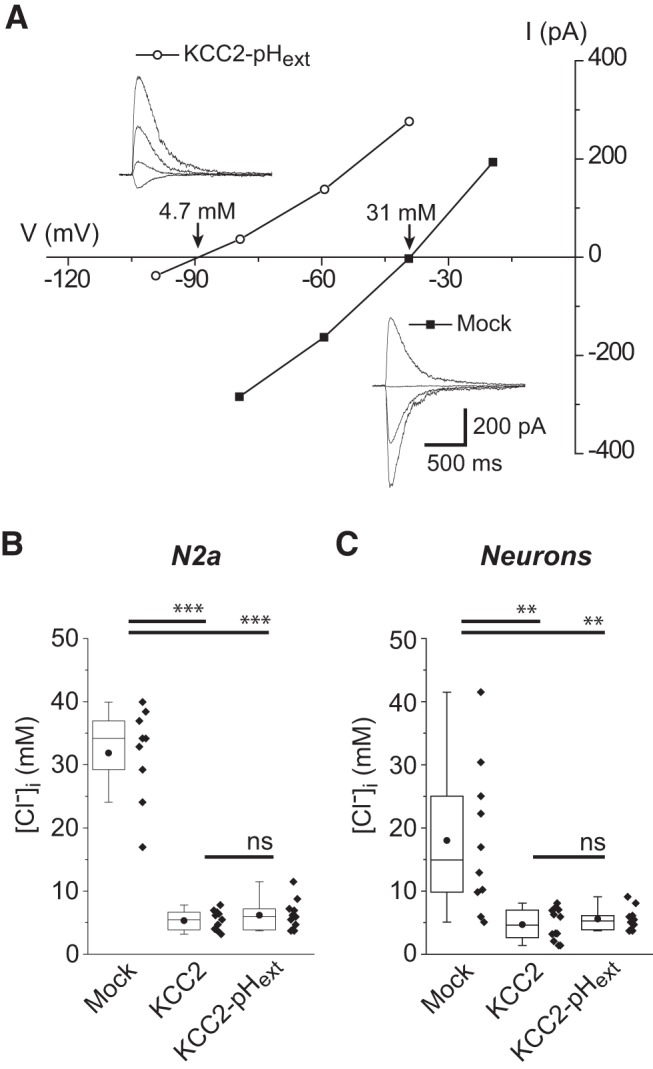
Recombinant KCC2-pH_ext_ is functional. ***A***, Traces show representative currents induced by focal applications of glycine to N2a cells expressing KCC2-pH_ext_ or eGFP (mock) at different voltage steps. In the I-V plot, GlyR-mediated current amplitudes were plotted against holding membrane potential. The current intercepts the voltage axis at EGly (–89 and –41 mV for KCC2-pH_ext_ and eGFP, respectively). The numbers above interception points indicated quantified using the Nernst equation [Cl^–^]_i_ values. ***B***, Tukey boxplots of the calculated [Cl^–^]_i_ values measured as shown in ***A*** for N2a cells expressing KCC2-pH_ext_, eGFP (mock), or KCC2-IRES-GFP (KCC2). Pooled data from four cultures, five to eight cells per culture and condition. ***, *p* < 0.001; ns, nonsignificant; one-way ANOVA and *post hoc* Tukey test (see Table 1 for details). ***C***, Calculated [Cl^–^]_i_ from E_GABAA_ measurements in 8- to 9-DIV neurons expressing KCC2-pH_ext_, eGFP (mock), or KCC2-IRES-GFP (KCC2). Eight cultures, two to three neurons per culture and condition. **, *p* < 0.01; ns, nonsignificant; one-way ANOVA.

To examine whether the insertion of the pHluorin tag may compromise KCC2 function, we coexpressed the KCC2-pH_ext_ with glycine receptor-channels (GlyR) in N2a cells and recorded the reversal potential of GlyR (EGly), which is principally determined by [Cl^-^]_i_, using gramicidin-perforated patch-clamp technique. The cells expressing KCC2-pH_ext_ were recognized through the weak fluorescence emitted by the pHluorin. For comparison, we used cells expressing eGFP only (mock) and cells expressing nontagged KCC2 subcloned in pIRES-GFP vector (Chudotvorova et al., 2005). Figure 2*A* shows an example of recording obtained for a KCC2-pH_ext_ or mock-transfected cell. We found that the [Cl^-^]_i_ calculated from EGly using the Nernst equation was much lower in KCC2-pH_ext_–expressing cells than in mock-transfected cells (statistically significant difference, *p* = 3.75 × 10^−13^; Table 1) and was similar to the one recorded in cells expressing nontagged KCC2 (*p* = 0.89, Fig. 2*B* and Table 1). These data indicated that KCC2-pH_ext_ overexpressed in a heterologous expression system was functional and did not differ from nontagged KCC2 in its ability to extrude Cl^-^.

In its native neuronal environment, KCC2 interacts with at least eight partners (Medina et al., 2014); some of them are neuron restricted and control KCC2 activity (*e.g.*, Neto2; Ivakine et al., 2013). To assess whether KCC2-pH_ext_ is active when expressed in neurons, we overexpressed the construct in cultured immature hippocampal neurons at developmental stage 8–9 DIV, characterized by low functional activity of endogenous KCC2 and relatively high level of [Cl^-^]_i_ (Chudotvorova et al., 2005; Pellegrino et al., 2011; Friedel et al., 2015). The measurements of the reversal potential of Cl^-^ permeable GABA_A_ receptor-channel (GABA_A_R) responses revealed statistically significant decrease of [Cl^-^]_i_ in neurons expressing KCC2-pH_ext_ compared to mock-transfected neurons (Fig. 2*C* and Table 1). [Cl^-^]_i_ in neurons expressing KCC2-pH_ext_ was indistinguishable from that measured in neurons transfected with nontagged KCC2. Thus, KCC2-pH_ext_ effectively reduces [Cl^-^]_i_ when expressed in either a heterologous expression system or cultured hippocampal neurons.

### Surface labeling of KCC2-pH_ext_ mutants with deleted intracellular regions

**Figure 3.**
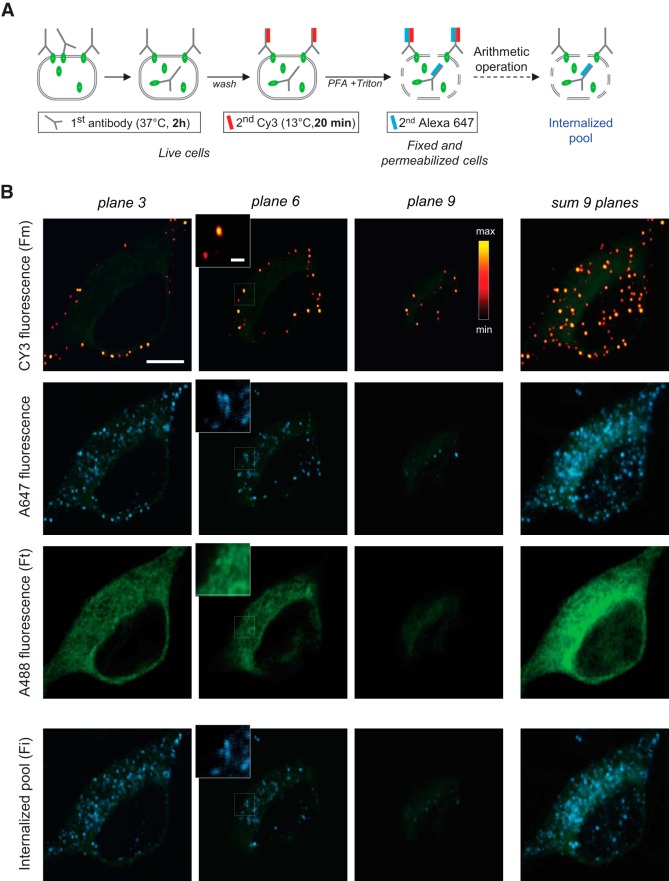
Visualization of surface expressed and internalized KCC2-pH_ext_ proteins using a live-cell immunolabeling protocol on cultured hippocampal neurons. ***A***, Scheme of the multistep immunolabeling protocol applied to 13 DIV neurons. First Ab, primary antibody; second Cy3, Cy3-conjugated secondary antibody; second Alexa 647, Alexa Fluor 647–conjugated secondary antibody; PFA, paraformaldehyde. The scheme does not include final steps of fixed and permeabilized cells labeled with mouse anti-GFP and anti-mouse Alexa Fluor 488 antibody [total protein pool (F_t_)]. ***B***, Representative images showing fluorescence emitted after staining with Cy3-conjugated secondary antibody [plasma membrane restricted pool (F_m_)]; images were pseudocolored using illustrated bicolor lookup table, first raw); Alexa Fluor 647–conjugated secondary antibody (internalized surface labeled molecules and portion of surface retained molecules, second raw); Alexa Fluor 488–conjugated secondary antibody (F_t_, third raw); internalized surface labeled signal obtained by arithmetic subtraction of first and second raw images (F_i_, fourth raw). Image columns illustrate fluorescent signals obtained at different *z*-planes or after arithmetic summation of nine planes as indicated. The neuronal shape (Alexa Fluor 488 fluorescence) is shown in light green in each image for reference. Insets illustrate indicated portions of images at higher zoom. Scale bars: 8 µm (main image), 1 µm (insets).

**Figure 4.**
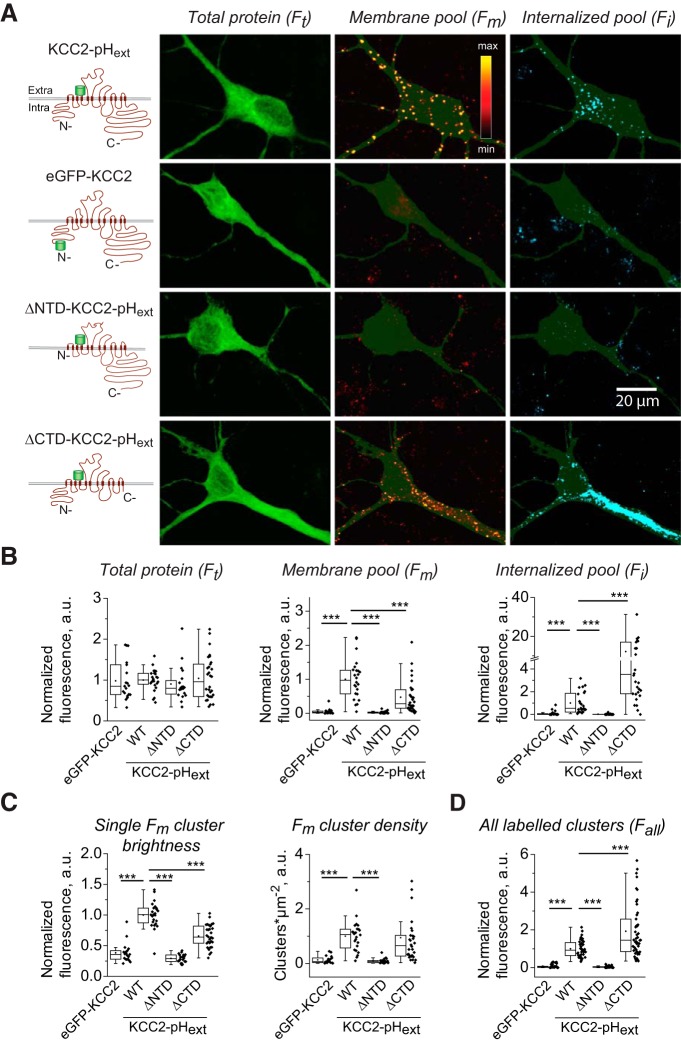
Surface expression of KCC2-pH_ext_ mutants with deleted intracellular N and C termini in cultured hippocampal neurons. ***A***, Schematic drawings of KCC2-pH_ext_ mutants and representative images showing fluorescence emitted after total protein staining (F_t_) by plasma membrane restricted pool (F_m_) and internalized pool of labeled molecules (F_i_). Images illustrate the fluorescence obtained after the summation of 10 *z*-planes acquired for each channel as described in Figure 3*B*. ***B–D***, Summary data of indicated morphometric parameters characterizing surface expression of mutants (values were normalized to mean KCC2-pH_ext_ in each experiment). The protocol used to determine the F_all_ depicted in ***D*** is described in Materials and Methods. Pooled data from four cultures, four to eight neurons per culture and condition. ***, *p* < 0.001, Mann–Whitney nonparametric test (see Table 2 for more details). Parameters of boxplots are the same as detailed in Figure 1.

Next we assessed the abundance of KCC2-pH_ext_, ΔNTD-KCC2-pH_ext_, and ΔCTD-KCC2-pH_ext_ in different cell compartments of 13 DIV hippocampal neurons using the previously described live-cell surface protein immunolabeling protocol (Fig. 3*A*; Friedel et al., 2015). Namely, we labeled the extracellular tag of KCC2-pH_ext_ using polyclonal rabbit anti-GFP antibody during 2 h on living neurons and under normal culturing conditions (37°C, 5% CO_2_). Presumably, at the end of this procedure, some of the labeled molecules remained on the cell surface, whereas others were internalized. The labeled molecules that were retained on the cell surface were visualized using secondary Cy3 conjugated anti-rabbit antibody applied to the living cells at low temperature (13°C), to prevent further protein internalization; the resulting fluorescence was identified as membrane fluorescence (F_m_). This live-cell labeling protocol revealed small (<0.5 µm) and distinct clusters of different intensity that were restricted to the cell surface, as it is illustrated with images taken at different *z*-planes (Fig. 3*A* and first raw images in Fig. 3*B*). These clusters resembled by their form and size the live-cell labeled clusters of another KCC2 construct harboring 3xFlag as an extracellular tag (Chamma et al., 2013). The second pool of molecules, corresponding to KCC2-pH_ext_ labeled and internalized, was visualized after *post hoc* fixation of the same cells and additional labeling with Alexa Fluor 647–conjugated anti-rabbit antibody. The Alexa Fluor 647–emitted fluorescence signal obtained was mainly restricted to the intracellular compartments, but also revealed partially the Cy3-positive clusters retained on the cell surface (Fig. 3*A* and second raw images in Fig. 3*B*). The “pure” internalized pool of fluorescence (F_i_) was then obtained after arithmetic subtraction of Alexa Fluor 647 and Cy3 images (Fig. 3*A* and fourth raw images in Fig. 3*B*). To quantify the total amount of overexpressed KCC2-pH_ext_, neurons were then labeled with mouse monoclonal anti-GFP antibody and revealed using Alexa Fluor 488 conjugated antibody (F_t_, total fluorescence intensity, third raw images in Fig. 3*B*). The specificity of KCC2-pH_ext_–related constructs surface labeling was verified using eGFP-KCC2 construct that carried a tag linked to the intracellular N terminus of KCC2. The surface immunolabeling of neurons expressing eGFP-KCC2 did not reveal regularly distributed Cy3 or Alexa Fluor 647–positive clusters. Only rare clusters of significantly weaker fluorescence intensity contributed to formation of the background signal (Fig. 4*A*, second images raw).

Although the four tested constructs (KCC2-pH_ext_, ΔNTD-KCC2-pH_ext_, ΔCTD-KCC2-pH_ext_, and eGFP-KCC2) were well expressed into neurons and showed similar intensities of F_t_, the amount of surface labeled fluorescent clusters strongly varied depending on the expressed molecule (Fig. 4*A*, *B* and Table 2). Unlike neurons expressing wild-type KCC2-pH_ext_, the cells with ΔNTD-KCC2-pH_ext_ mutant were not decorated with bright puncta, resulting in a significantly lower F_m_ (Fig. 4*A*, *B* and Table 2). The brightness and density of rare fluorescent F_m_ clusters detected on ΔNTD-KCC2-pH_ext_ neurons were significantly less than those of KCC2-pH_ext_ and were reminiscent of those characterizing the background staining of control neurons with eGFP-KCC2 (Fig. 4*C* and Table 2). As a consequence, the population data describing the intensity of F_m_ signal in individual neurons were significantly lower in ΔNTD-KCC2-pH_ext_, as compared to KCC2-pH_ext_, and did not differ from eGFP-KCC2–transfected cells (Fig. 4*B*, plot F_m_, and Table 2). Unlike ΔNTD-KCC2-pH_ext_, the neurons expressing ΔCTD-KCC2-pH_ext_ construct had clearly detectable clusters located in the plasma membrane (Fig. 4*A*, fourth images raw). The density of these clusters was similar to the one visualized on neurons with wild-type KCC2-pH_ext_, but their brightness was statistically significantly weaker (Fig. 4*C* and Table 2). The intensity of F_m_ emitted by ΔCTD-KCC2-pH_ext_ neurons was significantly lower as compared to wild-type KCC2-pH_ext_, but was significantly higher as compared to ΔNTD-KCC2-pH_ext_ neurons and control eGFP-KCC2-positive cells (Fig. 4*B*, plot F_m_, and Table 2).

**Table 2.** Statistical differences among the samples illustrated in Figure 4

Location	Data reference	Data structure	Type of test	Power
a	Fig. 4*B*, plot F_t_	Nonnormal distribution	Mann–Whitney *U* test	
	eGFP-KCC2 vs. WT			*U =* 185, *n* = 19,24; *p =* 0.30
	WT vs. ΔNTD			*U =* 161, *n* = 24,20; *p =* 0.06
	WT vs. ΔCTD			*U =* 357, *n* = 24,30; *p =* 0.97
	ΔNTD vs. ΔCTD			*U =* 274, *n* = 20,30; *p =* 0.72
b	Fig. 4*B*, plot F_m_	Nonnormal distribution	Mann–Whitney *U* test	
	eGFP-KCC2 vs. WT			*U =* 9, *n* = 19,24; *p =* 2.42 × 10^–10^
	WT vs. ΔNTD			*U =* 476, *n* = 24,20; *p =* 1.36 × 10^–11^
	WT vs. ΔCTD			*U =* 568, *n* = 24,30; *p =* 1.89 × 10^–4^
	ΔNTD vs. ΔCTD			*U =* 26, *n* = 20,30; *p =* 4.96 × 10^–10^
	eGFP-KCC2 vs. ΔNTD			*U =* 220, *n* = 19,20; *p =* 0.4
	eGFP-KCC2 vs. ΔCTD			*U =* 47, *n* = 19,30; *p =* 1.09 × 10^–6^
c	Fig. 4*B*, plot F_i_	Nonnormal distribution	Mann–Whitney *U* test	
	eGFP-KCC2 vs. WT			*U =* 420, *n* = 19,24; *p =* 2.28 × 10^–7^
	WT vs. ΔNTD			*U =* 476, *n* = 24,20; *p =* 1.36 × 10^–11^
	WT vs. ΔCTD			*U =* 106, *n* = 24,30; *p =* 2.86 × 10^–6^
	ΔNTD vs. ΔCTD			*U =* 8, *n* = 20,30; *p =* 2.84 × 10^–12^
	eGFP-KCC2 vs. ΔNTD			*U =* 230, *n* = 19,20; *p =* 0.24
	eGFP-KCC2 vs. ΔCTD			*U =* 14, *n* = 19,30; *p =* 2.58 × 10^–8^
d	Fig. 4*C*, single F_m_	Nonnormal distribution	Mann–Whitney *U* test	
	eGFP-KCC2 vs. WT			*U =* 14, *n* = 19,24; *p =* 1.27 × 10^–9^
	WT vs. ΔNTD			*U =* 447, *n* = 24,20; *p =* 7.95 × 10^–12^
	WT vs. ΔCTD			*U =* 647, *n* = 24,30; *p =* 5.59 × 10^–8^
	ΔNTD vs. ΔCTD			*U =* 14, *n* = 20,30; *p =* 2.16 × 10^–11^
	eGFP-KCC2 vs. ΔNTD			*U =* 242, *n* = 19,20; *p =* 0.15
	eGFP-KCC2 vs. ΔCTD			*U =* 70, *n* = 19,30; *p =* 1.08 × 10^–5^
e	Fig. 4*C*, F_m_ density	Nonnormal distribution	Mann–Whitney *U* test	
	eGFP-KCC2 vs. WT			*U =* 16, *n* = 19,24; *p =* 2.28 × 10^–9^
	WT vs. ΔNTD			*U =* 470, *n* = 24,20; *p =* 1.58 × 10^–10^
	WT vs. ΔCTD			*U =* 452, *n* = 24,30; *p =* 0.11
	ΔNTD vs. ΔCTD			*U =* 33, *n* = 20,30; *p =* 2.25 × 10^–9^
	eGFP-KCC2 vs. ΔNTD			*U =* 206, *n* = 19,20; *p =* 0.66
	eGFP-KCC2 vs. ΔCTD			*U =* 58, *n* = 19,30; *p =* 3.36 × 10^–6^
f	Fig. 4*D*	Nonnormal distribution	Mann–Whitney *U* test	
	eGFP-KCC2 vs. WT			*U =* 0, *n* = 33,40; *p =* 2.64 × 10^–13^
	WT vs. ΔNTD			*U =* 1480, *n* = 40,37; *p =* 4.69 × 10^–14^
	WT vs. ΔCTD			*U =* 538, *n* = 40,46; *p =* 9.56 × 10^–4^
	ΔNTD vs. ΔCTD			*U =* 0, *n* = 37,46; *p =* 6.56 × 10^–15^
	eGFP-KCC2 vs. ΔNTD			*U =* 719, *n* = 33,37; *p =* 0.2
	eGFP-KCC2 vs. ΔCTD			*U =* 0, *n* = 33,46; *p =* 4.65 × 10^–14^

The decreased surface expression of ΔNTD-KCC2-pH_ext_ and ΔCTD-KCC2-pH_ext_ mutants could be the result of reduced surface delivery of the molecules or enhanced internalization processes. Analysis of the fluorescence intensity of immunolabeled and internalized clusters revealed no detectable F_i_ signal in neurons expressing ΔNTD-KCC2-pH_ext_ mutant (Fig. 4*A*, *B*), and thus, indicated that the deletion of N terminus prevented delivery of the KCC2 into the plasma membrane. In contrast to neurons with ΔNTD-KCC2-pH_ext_, the cells expressing ΔCTD-KCC2-pH_ext_ construct exhibited >10-fold higher rate of internalization (statistically significant difference, *p* = 2.86 × 10^−6^, Fig. 4*B*, plot F_i_, and Table 2).

The decreased surface delivery of ΔNTD-KCC2-pH_ext_ mutant was further confirmed in another set of experiments with modified live staining protocol (see Materials and methods for details) designed to visualize all molecules labeled on the cell surface during a 2-h period (Fig. 4*D*). Any labeled clusters were detected on ΔNTD-KCC2-pH_ext_–positive neurons; the median value of F_all_ signal measured on ΔNTD-KCC2-pH_ext_ cells was >20-fold lower than that of wild-type KCC2-pH_ext_ neurons (0.04 and 0.92, respectively, *p* = 2.64 × 10^−13^; Fig. 4*D* and Table 2). Moreover, the distribution of the F_all_ values in ΔNTD-KCC2-pH_ext_ neurons was similar to that measured in eGFP-KCC2-positive cells (nonsignificant difference, *p* = 0.2; Fig. 4*D* and Table 2). Unlike ΔNTD-KCC2-pH_ext_, the F_all_ signal in ΔCTD-KCC2-pH_ext_–positive neurons was well detectable and was even significantly stronger than that in wild-type KCC2-pH_ext_ (correspondent medians were 1.45 and 0.92, p = 9.56 × 10^−4^; Fig. 4*D* and Table 2). We concluded, therefore, that the deletion of the N terminus of KCC2-pH_ext_ fully abolished plasmalemmal delivery of the protein, whereas a construct composed only of the N terminus and transmembrane regions was well delivered to the cell surface. Once expressed into the plasma membrane, ΔCTD-KCC2-pH_ext_ proteins were rapidly internalized, which accounted for the twofold lower amount of F_m_ and higher amount of F_i_ compared with wild-type KCC2-pH_ext_. Taken together, these data indicated that N terminus is indispensable for surface delivery of KCC2-pH_ext_.

### Surface expression of KCC2-pH_ext_ mutants in heterologous cell lines

The neuronal environment is crucial for KCC2 function and regulation, and Li et al. (2007) found that ΔNTD-KCC2 was expressed in the plasma membrane of HEK293 cells. Therefore, we asked whether the surface delivery of ΔNTD-KCC2-pH_ext_ mutant is also compromised in cell lines. We examined the expression of nonmutated KCC2-pH_ext_, ΔNTD-KCC2-pH_ext_, and ΔCTD-KCC2-pH_ext_ mutants in two different cell lines, neuron-derived N2a and nonneuronal HEK293 cells.

**Figure 5.**
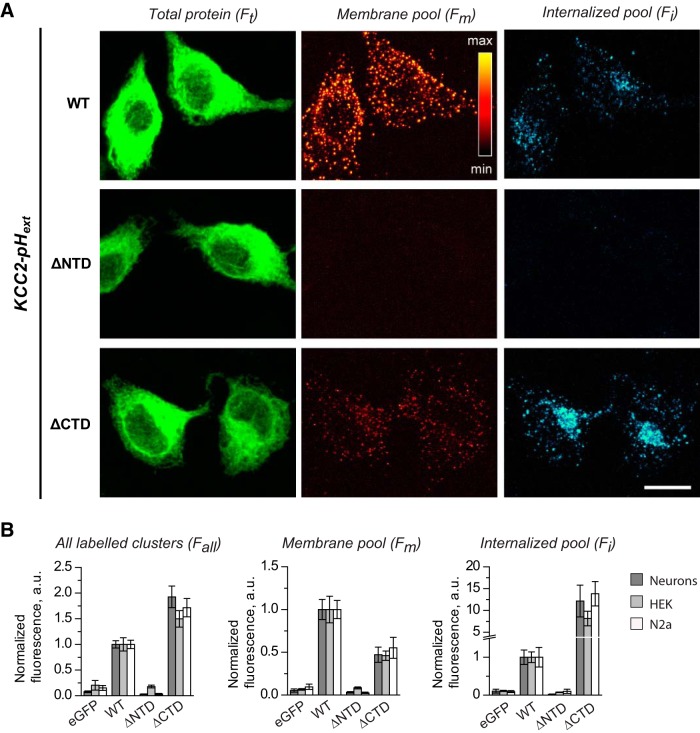
Properties of wild-type KCC2-pH_ext_ (WT) and ΔNTD- and ΔCTD-KCC2-pH_ext_ mutants are not restricted to neuronal environment. ***A***, Representative images of N2a cells showing total protein fluorescence (F_t_), plasma membrane staining (F_m_), and internalized fluorescence (F_i_) of indicated constructs in the same experimental paradigm as depicted in Figure 4. Scale bar, 20 µm. ***B***, Comparison of morphometric parameters characterizing surface expression of mentioned mutants in neurons, HEK293 cells, and N2a cells. Plots showing mean ± SEM of indicated values that were normalized to KCC2-pH_ext_. Pooled data from four experiments, 20–25 cells per experiment and condition. Statistical significance of differences between columns is shown in Table 1. Note that, as with neurons, in both cell lines the deletion of the N terminus of KCC2-pH_ext_ fully abolished the mutant’s surface expression, whereas the deletion of the C terminus facilitated plasma membrane surface delivery. Once delivered to the plasma membrane, ΔCTD-KCC2-pH_ext_ mutants were rapidly internalized. As a consequence, the amount of surface expressed ΔCTD-KCC2-pH_ext_ relative to wild-type KCC2-pH_ext_ was significantly lower in both HEK293 and N2a cells, whereas internalized pool of the mutant was 7- to 15-fold stronger in these cells, respectively.

**Figure 6.**
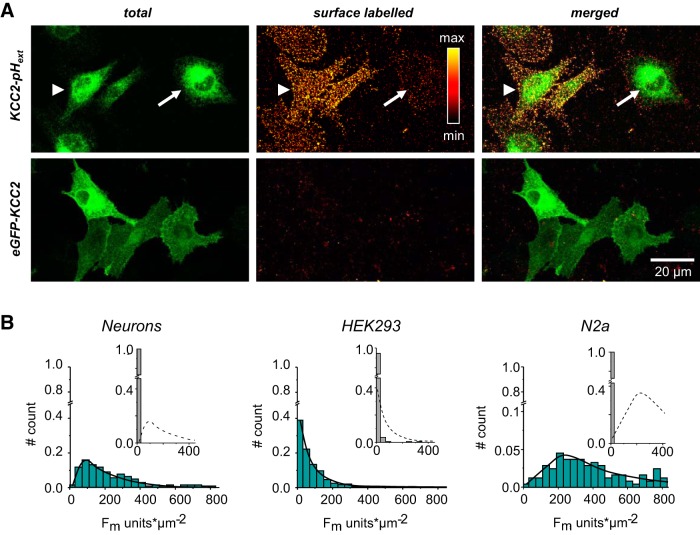
High variability in the surface expression of WT-KCC2-pH_ext_ in HEK293 cells. ***A***, Representative images of HEK293 cells illustrating variability in surface labeling of WT-KCC2-pH_ext_ (top). Although two HEK293 cells (arrowhead and arrow) show similar amounts of total expressed WT-KCC2-pH_ext_, the levels of surface labeled proteins differ in these cells. The bottom panel illustrates negative control surface labeling of eGFP-KCC2 construct with intracellularly located tag. ***B***, Distribution histograms characterizing surface labeling (F_all_) of WT-KCC2-pH_ext_ protein expressed in neurons, HEK293 cells, or N2a cells. Insets show the F_all_ distribution for the control eGFP-KCC2 construct. The dotted lines in insets reproduce the distribution profile of KCC2-pH_ext_ for comparison. Note that the distribution profile for HEK293 cells transfected with KCC2-pH_ext_ is different than that of neurons and N2a, with predominance of cells with low F_all_. Six experiments, 25 analyzed cells per experiment.

As in neurons, we found that in both cell lines the deletion of the N terminus of KCC2-pH_ext_ fully abolished the mutant’s surface expression, whereas the ΔCTD-KCC2-pH_ext_ construct composed of N terminus and transmembrane regions was well labeled on the cell surface (Fig. 5*A*). The relative intensities of F_m_ and F_i_ signals in HEK293 and N2a cell lines expressing ΔCTD-KCC2-pH_ext_ mutant resembled those analyzed in neurons (Fig. 5*B* and Table 3). We concluded, therefore, that the N terminus is essential for the plasmalemmal delivery of KCC2-pH_ext_ regardless of the expression system. Of note, we found that wild-type KCC2-pH_ext_ was labeled on the cell surface of 98% of studied neurons and 99% of N2a cells but only 62% of HEK293 cells (Fig. 6), indicating a possible difference in the mechanisms controlling KCC2 surface delivery in N2a and HEK293 cell lines.

**Table 3.** Statistical differences among the samples illustrated in Figure 5

Location	Data reference	Data structure	Type of test	Power
a	Fig. 5*B*, F_all_ HEK	Nonnormal distribution	Mann–Whitney *U* test	
	eGFP-KCC2 vs. WT			*U =* 995, *n* = 70,109; *p =* 2.18 × 10^–19^
	WT vs. ΔNTD			*U =* 11412, *n* = 109,117; *p =* 2.48 × 10^–29^
	WT vs. ΔCTD			*U =* 4182, *n* = 109,106; *p =* 4.32 × 10^–4^
	ΔNTD vs. ΔCTD			*U =* 1015, *n* = 117,106; *p =* 2.36 × 10^–33^
b	Fig. 5*B*, F_all_ N2a	Nonnormal distribution	Mann–Whitney *U* test	
	eGFP-KCC2 vs. WT			*U =* 16, *n* = 23,24; *p =* 1.13 × 10^–10^
	WT vs. ΔNTD			*U =* 552, *n* = 24,23; *p =* 1.24 × 10^–13^
	WT vs. ΔCTD			*U =* 173, *n* = 24,27; *p =* 0.004
	ΔNTD vs. ΔCTD			*U =* 9, *n* = 23,27; *p =* 1.80 × 10^–12^
c	Fig. 5*B*, F_m_ HEK	Nonnormal distribution	Mann–Whitney *U* test	
	eGFP-KCC2 vs. WT			*U =* 1076, *n* = 61,109; *p =* 8.23 × 10^–15^
	WT vs. ΔNTD			*U =* 10352, *n* = 109,117; *p =* 1.88 × 10^–17^
	WT vs. ΔCTD			*U =* 6428, *n* = 109,106; *p =* 0.15
	ΔNTD vs. ΔCTD			*U =* 2320, *n* = 117,106; *p =* 2.34 × 10^–17^
d	Fig. 5*B*, F_m_ N2a	Nonnormal distribution	Mann–Whitney *U* test	
	eGFP-KCC2 vs. WT			*U =* 11, *n* = 23,24; *p =* 2.42 × 10^–11^
	WT vs. ΔNTD			*U =* 546, *n* = 24,23; *p =* 3.72 × 10^–12^
	WT vs. ΔCTD			*U =* 679, *n* = 24,38; *p =* 0.0010
	ΔNTD vs. ΔCTD			*U =* 33, *n* = 23,38; *p =* 2.86 × 10^–12^
e	Fig. 5*B*, F_i_ HEK	Nonnormal distribution	Mann–Whitney *U* test	
	eGFP-KCC2 vs. WT			*U =* 1356, *n* = 61,109; *p =* 2.40 × 10^–11^
	WT vs. ΔNTD			*U =* 10638, *n* = 109,117; *p =* 3.26 × 10^–14^
	WT vs. ΔCTD			*U =* 2362, *n* = 109,106; *p =* 5.77 × 10^–15^
	ΔNTD vs. ΔCTD			*U =* 479, *n* = 117,106; *p =* 1.19 × 10^–32^
f	Fig. 5*B*, F_i_ N2a	Nonnormal distribution	Mann–Whitney *U* test	
	eGFP-KCC2 vs. WT			*U =* 41, *n* = 23,24; *p =* 3.03 × 10^–8^
	WT vs. ΔNTD			*U =* 548, *n* = 24,23; *p =* 1.49 × 10^–12^
	WT vs. ΔCTD			*U =* 142, *n* = 24,38; *p =* 1.62 × 10^–6^
	ΔNTD vs. ΔCTD			*U =* 11, *n* = 23,38; *p =* 1.04 × 10^–14^

### Biotinylation of KCC2 mutants

**Figure 7.**
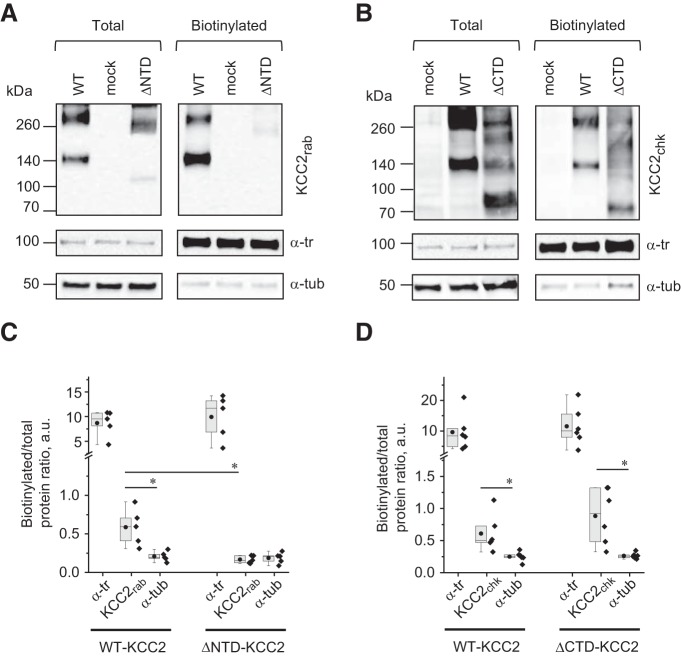
Surface biotinylation of nontagged KCC2 mutants. ***A***, Representative Western blots of total extracts (left) and biotinylated fractions (right) of N2a cells transfected with wild-type KCC2 (WT), pcDNA3.1 (mock), and ΔNTD-KCC2 (ΔNTD). Detection with anti-KCC2 rabbit (KCC2_rab_) antibody recognizing C terminus of KCC2. Anti–α-transferrin receptor antibody (α-tr) and anti–α-tubulin antibody (α-tub) were used to normalize KCC2 signals and reveal the plasma membrane selectiveness and background of the biotinylation procedure. ***B***, Western blot of N2a cells expressing pcDNA3.1 (mock), wild-type KCC2 (WT), and ΔCTD-KCC2 (ΔCTD). Detection with anti-KCC2 chicken (KCC2_chk_) antibody recognizing N terminus of KCC2. ***C***, Summary data of surface biotinylation rates (biotinylated/total ratio) for α-tr, KCC2, and α-tub proteins in samples extracted from cells transfected with either WT-KCC2 or ΔNTD-KCC2 and revealed as described in ***A***. *, *p* < 0.05, *n* = 5, nonparametric Wilcoxon matched pairs test. ***D***, Summary data of surface biotinylation rates for α-tr, KCC2, and α-tub proteins in samples extracted from cells transfected with either WT-KCC2 or ΔCTD-KCC2 and revealed as described in ***B***. *, *p* < 0.05, *n* = 6, nonparametric Wilcoxon matched pairs test. Parameters of boxplots are the same as detailed in Figure 1.

To exclude a possible contribution of the extracellular tag to the mutant surface delivery and internalization, we analyzed the surface expression of nontagged wild-type KCC2, ΔNTD-KCC2, and ΔCTD-KCC2 proteins using biotinylation assay in N2a cells followed by SDS-PAGE separation and Western blotting (Fig. 7). As in the Western blot analysis depicted in Figure 1, the expression of ΔNTD-KCC2 mutant was visualized using anti-KCC2_rab_ antibody recognizing the C terminus of KCC2 (Fig. 7*A*), whereas the expression of ΔCTD-KCC2 mutant was detected with anti-KCC2_chk_ antibody recognizing the N terminus of the transporter (Fig. 7*B*). In the same samples, we also characterized the cellular expression and surface biotinylation level of the transmembrane α-transferrin receptor (α-tr) and cytoplasmic α-tubulin (α-tub). α-tr labeling was used as a positive control for biotinylation efficacy and loading of total and biotinylated protein fractions. α-tub, an intracellular protein that should not be biotinylated, was used as a negative control to determine the level of inevitable leakage of the biotinylation compound into the intracellular environment. Special attention was given to the improvement of the experimental protocol, with the purpose of decreasing biotin leakage (see Materials and Methods for details). We quantified the intensity of each band and expressed the results as the ratio of biotinylated to total protein for α-tr, α-tub, and KCC2 (Fig. 7*C*, *D*). As illustrated in Figure 7*A* and quantified in Figure 7*C*, the biotinylated/total protein ratio was significantly lower in neurons expressing ΔNTD-KCC2 compared with neurons expressing WT-KCC2. Moreover, consistent with experiments involving ΔNTD-KCC2-pH_ext_ (Fig. 5), the biotinylation level of ΔNTD-KCC2 was indistinguishable from that of leakage biotinylation revealed using α-tub antibody (*p* = 0.69; Fig. 7*C* and Table 4). Unlike the ΔNTD-KCC2 mutant, the level of the surface labeling of ΔCTD-KCC2 mutant was not statistically different from the labeling of WT-KCC2 (*p* = 0.92; Fig. 7*B* and Table 4) and was significantly higher than the level of surface unrelated labeling of α-tub in the same cell sample (*p* = 0.02; Fig. 7*D* and Table 4). We concluded, therefore, that the truncation of the N terminus of naive KCC2 prevented plasmalemmal expression of the transporter, whereas the truncation of the C terminus did not affect this process.

**Table 4.** Statistical differences among the samples illustrated in Figure 7

Location	Data reference	Data structure	Type of test	Power
a	Fig. 7*C*	Nonnormal distribution	Wilcoxon matched pairs test	
	WT, KCC2rab vs. WT, Tub			*z =* 1.89, *n* = 5; *p =* 0.031
	WT, KCC2rab vs. ΔNTD, KCC2rab			*z =* 1.89, *n* = 5; *p =* 0.031
	ΔNTD, KCC2rab vs. ΔNTD, Tub			*z =* –0.27, *n* = 5; *p =* 0.69
b	Fig. 7*D*	Nonnormal distribution	Wilcoxon matched pairs test	
	WT, KCC2chk vs. WT, Tub			*z =* 2.10, *n* = 6; *p =* 0.016
	WT, KCC2chk vs. ΔCTD, KCC2chk			*z =* –1.26, *n* = 6; *p =* 0.92
	ΔCTD, KCC2chk vs. ΔCTD, Tub			*z =* –2.10, *n* = 6; *p =* 0.016

## Discussion

**Figure 8.**
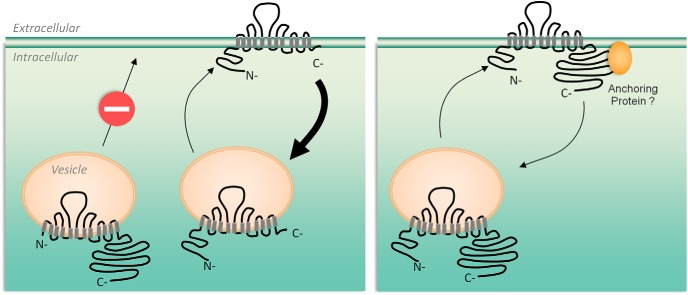
Scheme of the surface expression of different KCC2 mutants. Deletion of the N terminus abolishes plasmalemmal delivery of the transporter, whereas deletion of the C terminus does not interrupt this process. Thus, the N terminus is indispensable for KCC2’s surface delivery. The mutant with deleted C terminus is internalized more effectively than the wild-type KCC2 (left). We postulate that wild-type KCC2 is stabilized in the plasma membrane using a putative anchoring protein that interacts with the C terminus of the transporter (right).

In the present work, we demonstrated that KCC2-pH_ext_ is a suitable and useful tool to assess the membrane expression of exogenous KCC2 proteins in neurons and heterologous systems. We found that the N terminus is required for KCC2 insertion into the plasma membrane, regardless of the expression system, whereas the C terminus is critical for the membrane stability of KCC2. Our results suggest that cargo proteins responsible for KCC2 trafficking to the plasma membrane are interacting with regions other than the C terminus and highlight the N terminus of KCC2 as a critical element for this mechanism (Fig. 8).

We took advantage of the KCC2-pH_ext_ construct to analyze the role of the intracellular tails in the plasma membrane expression and internalization of KCC2. Although questions about the role of diverse mutations and structural elements on KCC2 surface expression are of primary importance, the relatively low percentage of endogenous KCC2 expressed at the plasma membrane and the absence of specific antibody recognizing extracellular domains of the transporter make these questions challenging. To circumvent these limitations, a number of KCC2 chimeras with tags in the extracellular loops were created and successfully used to characterize the surface expression of different mutants (Zhao et al., 2008; Acton et al., 2012; Chamma et al., 2013; Weber et al., 2014). Along this line, KCC2-pH_ext_, was previously used to characterize the surface expression of KCC2 mutants associated with human idiopathic generalized epilepsy (Kahle et al., 2014) and describe the functional significance of KCC2’s Thr906 and Thr1007 phosphorylation sites (Friedel et al., 2015). In the present study, we performed a detailed analysis of the biochemical and biophysical properties of KCC2-pH_ext_. We demonstrated that the insertion of the pHluorin tag does not affect the migration pattern of KCC2 and preserves its ion transport ability. More importantly, the pHluorin tag did not modify the migration pattern of mutants with deleted N and C termini and yielded discoveries about novel roles of intracellular tails in surface expression of KCC2.

Our main discovery is the critical role of the N terminus for the surface delivery of KCC2. This finding was unexpected, as a previous study illustrated the effective biotinylation (*e.g.*, surface expression) of ΔNTD-KCC2 in HEK293 cells (Li et al., 2007). The cited work was, however, qualitative and did not provide quantitative analysis or take into account controls of the biotin leakiness in cells expressing KCC2 and ΔNTD-KCC2. So far, no other studies have been performed to address the question of the role of the N terminus for proper surface delivery of KCC2. We have found that decreased surface expression of ΔNTD-KCC2-pH_ext_ mutant is the result of perturbed protein surface delivery, whereas decreased surface expression of ΔCTD-KCC2-pH_ext_ mutant is consequent to an enhanced protein internalization rate. These results were further confirmed using a biotinylation approach and nontagged KCC2 constructs.

What clues explain the default of ΔNTD-KCC2 expression at the plasma membrane? Our results showing the expected molecular weight of the monomeric form of ΔNTD-KCC2 mutant suggest that the protein is at least correctly translated and are consistent with a previous study (Horn et al., 2010). Also, the authors found that ΔNTD-KCC2 interacts with the neuronal cytoskeleton-associated protein 4.1N similarly to the wild-type KCC2 and therefore suggested that ΔNTD-KCC2 mutant is correctly folded. In that case, one can suggest that the deletion of the N terminus perturbs the interaction of KCC2 with surface-delivery machinery without affecting structural integrity of the mutant. However, our finding of the modified monomer/total protein ratio does not exclude possible modifications in the tertiary/quaternary structure of the ΔNTD-KCC2 protein that could cause the mutant to be stacked at one of the multiple checkpoints of the secretory pathway. Future studies are required to characterize in more detail the structural changes associated with the deletion of the N terminus of KCC2.

We further highlighted the critical role of the N terminus for KCC2 surface delivery using two constructs composed only of N terminus and transmembrane regions (ΔCTD-KCC2-pH_ext_ and ΔCTD-KCC2 mutants). Both mutants were effectively inserted into the plasma membrane. This observation provides a novel perception of the structural role of the C terminus in KCC2’s function and indicates that the C terminal domain is not required for the surface insertion of the transporter. However, after effective delivery to the plasma membrane, the ΔCTD-KCC2-pH_ext_ mutant was rapidly internalized, suggesting an important role of the C terminus for KCC2 stabilization into the plasma membrane. An alternative explanation could be that because of a compromised structure and folding, the protein undergoes rapid internalization to be further degraded. This suggestion is in agreement with the consistent observation of a smear-like pattern of migration for ΔCTD-KCC2-pH_ext_ and ΔCTD-KCC2 mutants. Yet the mechanisms controlling the internalization and degradation of KCC2 are out of the scope of the present work and constitute a crucial subject for further studies.

Understanding the structure–function organization of KCC2 transporter is a priority in the field, as KCC2 is a putative target for development of therapeutic treatments (Kahle et al., 2013; Löscher et al., 2013; Medina et al., 2014). To date, most reported functionally important KCC2 regulatory sites are located on the long intracellular C terminus (Lee et al., 2007, 2010; Li et al., 2007; Rinehart et al., 2009; Acton et al., 2012; Kahle et al., 2014; Puskarjov et al., 2014; Weber et al., 2014). The structure–function importance of the intracellular N terminus of KCC2 is much less studied. A recent work suggested the importance of KCC2 N terminus domain for the neuroprotection (Winkelmann et al., 2015). The N terminus of KCC2 also carries several putative regulatory sites, including phosphorylatable serine residues (Weber et al., 2014) and the Ste20-related proline alanine-rich kinase/oxidative stress response-1 (SPAK/OSR1) binding motif (Uvarov et al., 2007); however, their functional importance remains obscure. The present work provides an important clue for further research directed to describe the mechanisms of KCC2 surface insertion and the precise role of the N terminus domain in this process. In addition to the surface insertion mechanism, we have noticed the importance of the N terminus for KCC2 expression into distal dendrites. Indeed, the truncation of the N terminus of KCC2-pH_ext_ resulted in decreased dendritic expression of the transporter, whereas the construct composed of only the N terminus and transmembrane domains showed twofold higher dendritic expression. These results are in agreement with similar findings reported by Winkelmann et al. (2015). Although identification of the mechanisms involved in the decreased expression of ΔNTD-KCC2 in the distal dendritic compartment was not in the scope of the present study, our findings highlight one more potential structure–function role for the N terminus region that should be taken into account in future experiments.

The KCC2 mutant with deleted N terminus is widely used as a molecular tool in studies to characterize mechanisms of regulatory actions of KCC2 (Li et al., 2007; Bortone and Polleux, 2009; Horn et al., 2010; Gauvain et al., 2011; Fiumelli et al., 2013; Winkelmann et al., 2015). In particular, the experiments using ΔNTD-KCC2 mutant served as a key argument in the hypothesis of the structural (ion transport–independent) mechanism of KCC2’s regulatory action (Li et al., 2007; Horn et al., 2010; Fiumelli et al., 2013). Our finding does not place this hypothesis in question, as ΔNTD-KCC2 possesses the putative region on the C terminus mediating the trophic action of KCC2 (Li et al., 2007), and overexpression of only intracellular C terminus exerted a similar regulatory action (Fiumelli et al., 2013); however, it should be considered for future work, suggesting proper dendritic delivery and surface expression of the transporter.

In conclusion, our results support a novel view of the role of the N terminus domain in the control of KCC2 surface expression. Because KCC2 is a putative target for novel therapeutic treatments, our findings highlight the N terminus as a crucial target for strategies designed to enhance KCC2’s surface delivery.

## Synthesis

Reviewing Editor: Quentin Pittman, University of Calgary Decisions are customarily a result of the Reviewing Editor and the peer reviewers coming together and discussing their recommendations until a consensus is reached. When revisions are invited, a fact-based synthesis statement explaining their decision and outlining what is needed to prepare a revision will be listed below. The following reviewer(s) agreed to reveal their identity: Melanie Woodin, Christoph Fahlke.

The reviewers agree that the authors are addressing an important question in KCC2 biology: what is the function of the intracellular N-terminus? In general the experiments appear to be well conducted, with appropriate controls. The primary conclusion is that the N-terminus is required for surface membrane expression, and this finding will make an important contribution to the field of KCC2 biology.

Major

The reviewers were impressed with many of your experiments and claims. However, both reviewers feel that the attempts to examine oligomeric structure, while an obvious eventual goal, are outside of the scope of the study and in the current form are unconvincing due to smearing of the gels due to possible poor solubilization. Furthermore, they suggest that a proper investigation of this is challenging and will require many more experiments. Thus they suggest that you remove at least fig 7E and also address the smearing in 7B.

Further specific points that must be addressed

• Figure 1C. The authors claim that the N-terminal regulates membrane insertion (and not protein expression), thus they should quantify the Western blot data (normalized to loading tubulin). In addition, they should indicate how many independent biological replicates were performed. The authors' state in the Methods (line ~215) that the upper bands are agglomerates corresponding to dimer or oligomers, but it is possible these bands are aggregates of protein fragments. This is an important consideration because it appears that these higher MW bands are more prominent in the N-term deletion. Lastly, methods related to Western blots should explain the sample preparation (e.g. temperature).

• Figure 1D-F. These data demonstrate that there is no difference in expression of the N-terminal deleted KCC2 in the soma, but there is a significant decrease in the dendrites. Taken together, this would suggest an overall decrease in expression of N-term KCC2 throughout the neuron. Thus the authors should report total fluorescence intensity of the neuron, or quantify the Western blot as suggested above. N values should be clearly stated for each condition.

• The statement on line 64 ‘C-terminus does not contribute to the plasmalemmal delivery’ may be an overstatement of the current results. While the significant decrease in surface expression in Figure 4 B could be due to the increase in internalization, it does not discount a role for the C-terminus in delivery of the transporter to the surface membrane.

• Figure 7A. There appears to be a significant reduction in the protein expression of the N-terminal deleted KCC2 monomer (relating again to my concern above about protein expression). Based on this apparent decreased monomer expression it is not surprising that there is nothing biotinylated.

• Figure 7B. There is extensive smearing in the C-terminal deleted lanes (both the total input and biotinylated). What accounts for this?

• Figure 7E. According to Figure 5 there is no N-term deleted KCC2 in the surface membrane of HEK293 cells, so it is not clear how in this experiment (7E) N-term deleted KCC2 was extracted from membranes of HEK293 cells.

• Figure 7E the two lanes in the blot should be labelled and/or described in the legend. I am assuming that the band on the right in the native PAGE. In the WT KCC2 a band is present, but in the N-term deletion a smear is present (with no associated band). Smearing in native bands is often attributed to protein aggregation, protein fragments, or incomplete solubilization. Line 450 states ‘absence of aggregates of N-term deletion in Native page’ and I am not sure that this statement is justified.

Additional points:

• The manuscript should be edited for grammar and appropriate use of the English language.

• The first paragraph of the discussion very specifically addresses opposing results obtained in another paper. It was suggested that having a more general first paragraph before comparing and contrasting with previously published literature.

• The external pHfluorin tagging as tool for studying surface membrane insertion was appreciated. However, an obvious caveat of this approach is that the tagged protein might have altered subcellular localization of KCC2. The biotinylation experiments in Fig. 7 clearly demonstrate that this is not the case or at least that the conclusion of the manuscript are not affected. However, authors should address this concern earlier in the manuscript

• N values should be clearly listed in the figure legends for each individual experiment.
